# Hierarchical Porous Activated Carbon Derived from Coconut Shell for Ultrahigh-Performance Supercapacitors

**DOI:** 10.3390/molecules28207187

**Published:** 2023-10-20

**Authors:** Yawei Wang, Yuhui Duan, Xia Liang, Liang Tang, Lei Sun, Ruirui Wang, Shunhang Wei, Huanan Huang, Pinghua Yang, Huanan Hu

**Affiliations:** 1 School of Chemistry and Chemical Engineering, Jiangxi Province Engineering Research Center of Ecological Chemical Industry, Jiujiang University, Jiujiang 332005, China; 20200205648@jju.edu.cn (X.L.); 20200200942@jju.edu.cn (L.T.); 20200205239@jju.edu.cn (L.S.); 6080129@jju.edu.cn (R.W.); huanghn@jju.edu.cn (H.H.); 6080021@jju.edu.cn (P.Y.); 2 Institute for Advanced Interdisciplinary Research (iAIR), University of Jinan, Jinan 250022, China; 202130222057@stu.ujn.edu.cn; 3 School of Mathematical Information, Shaoxing University, Shaoxing 312000, China; wsh@usx.edu.cn

**Keywords:** hierarchical porous activated carbon, coconut shells, ultrahigh-performance, KOH symmetric supercapacitors, zinc–ion hybrid supercapacitors

## Abstract

In this research, we successfully produced hierarchical porous activated carbon from biowaste employing one-step KOH activation and applied as ultrahigh-performance supercapacitor electrode materials. The coconut shell-derived activated carbon (CSAC) features a hierarchical porous structure in a honeycomb-like morphology, leading to a high specific surface area (2228 m^2^ g^−1^) as well as a significant pore volume (1.07 cm^3^ g^−1^). The initial test with the CSAC electrode, conducted in a 6 M KOH loaded symmetric supercapacitor, demonstrated an ultrahigh capacitance of 367 F g^−1^ at a current density of 0.2 A g^−1^ together with 92.09% retention after 10,000 cycles at 10 A g^−1^. More impressively, the zinc–ion hybrid supercapacitor using CSAC as a cathode achieves a high-rate capability (153 mAh g^−1^ at 0.2 A g^−1^ and 75 mAh g^−1^ at 10 A g^−1^), high energy density (134.9 Wh kg^−1^ at 175 W kg^−1^), as well as exceptional cycling stability (93.81% capacity retention after 10,000 cycles at 10 A g^−1^). Such work thus illuminates a new pathway for converting biowaste-derived carbons into materials for ultrahigh-performance energy storge applications.

## 1. Introduction

In order to address the escalating demand for energy, continuous advancements have been achieved in the domain of energy conversion and storage devices [1,2]. Supercapacitors, which may fill the gap between traditional capacitors and rechargeable batteries in terms of energy storage, have intrigued researchers much because of their high power density, excellent cycling stability, and swift charge–discharge speed [3,4,5]. Nevertheless, to meet the long endurance requirements of actual applications, supercapacitors are deemed to be designed with a high energy density. The voltage window (*V*) and the capacitance (*C*) of the supercapacitor can be elevated to increase the energy density (*E*) in accordance with the formula *E =* 1/2*CV*^2^ [6]. Generally, the working voltage depends on the stable electrochemical window of the electrolyte, whereas the capacitance is dictated by the physicochemical properties of electrode materials [7,8]. As a result, multiple supercapacitors are proposed to hasten the energy/power density ratio by creating new electrode/electrolyte materials and optimizing the design of supercapacitors [9,10].

Due to their high surface area and suitable porous structure, which are the primary factors affecting electrochemical energy storage in supercapacitors, hierarchical porous activated carbons (HPACs) have been acknowledged as active electrode materials in varying supercapacitors [11,12]. Electrolyte ions move swiftly from the macropores to the micropores through the mesoporous pathways in hierarchical porous structures, achieving the necessary adsorption of ions on the substantial specific surface area [13,14]. Thereby, developing HPACs with a large surface area has been considered an appealing choice for enhancing supercapacitors’ ability to store energy [15]. Typically, HPACs are produced from fossil feedstocks or organic polymers using template or physicochemical activation methods [16,17,18,19]. However, the consumption of organic polymers, fossil fuels and templates unavoidably increases the price of HPACs synthesis, further hindering their broad use in supercapacitors. Biowaste materials, including pitaya peel [20], corncob [21], bio-oil [22], durian kernel [23] and sesame husk [24], have been employed to create HPACs on an industrial large-scale and also served as electrode materials in supercapacitors due to their unignorable benefit of low cost [25]. Among various biowaste-based supercapacitors, zinc–ion hybrid supercapacitors (ZHSs) directly utilize naturally abundant zinc foils as the anode, biowaste-derived carbons as the cathode materials and neutral electrolytes (ZnSO_4_ or Zn(CF_3_SO_3_)_2_) for a comparatively high working voltage (~1.8 V in the aqueous electrolyte) [26,27]. Therefore, such a ZHSs system, which inherits the merits of high-power supercapacitors and high-energy batteries, have gained extensive interest due to their superior energy/power ratio, dependable safety, low cost and high theoretical capacity (~820 mAh g^−1^) [28,29]. Leveraging the distinctive design of HPACs, derived from biowaste, as cathode materials can effectively meet the high energy storage performance and industrial-production demands of ZHSs in today’s market. For instance, Zhang et al. applied pencil-shaving derived porous carbon as a cathode material and the assembled ZHSs obtained a superior capacity of 183 mAh g^−1^ at 0.2 A g^−1^ in Zn(CF_3_SO_3_)_2_ electrolyte with 92.2% capacity retention after 10,000 cycles [30]. Chen et al. prepared N, O co-doped 2D carbon nanosheets from poplar wood and achieved a capacity of 111.0 mAh g^−1^ at 0.1 A g^−1^ in 2 M ZnSO_4_ electrolyte [31].

Herein, due to the enormous annual production of coconuts (~60 million tons worldwide), coconut shell was chosen in this study as the possible precursor for the large-scale manufacturing of HPAC and commercial application in energy storage devices. The coconut shell-derived carbon activated by KOH (CSAC) we have prepared features a honeycomb-like morphology, a large specific surface area and hierarchical porous structure. These attributes equip the CSAC with a high energy storage performance and enduring cyclability, performing well in both conventional symmetric and hybrid supercapacitors. Our findings underscore the potential of HPAC derived from the coconut shell as an exceptional electrode material for ultrahigh-performance supercapacitors.

## 2. Results and Discussion

**Figure 1:**
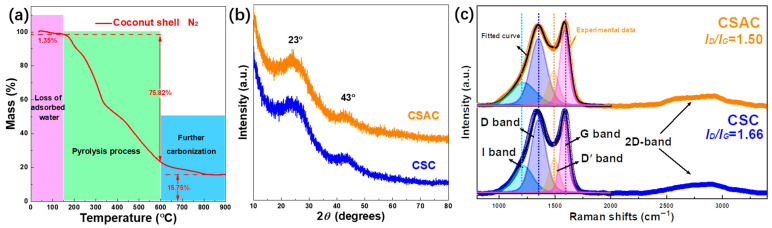
(**a**) TG curve of coconut shell under N_2_ atmosphere from room temperature to 900 °C, (**b**) XRD patterns and (**c**) Raman spectra of CSC and CSAC.

The thermogravimetric curve of the precursor in Figure 1a was procured under a nitrogen atmosphere to examine the carbonization of coconut shell. The TG curve appears to be roughly divided into three regions: the release of absorbed water causes the mass curve of a coconut shell to drop by around 1.35% in the purple low-temperature region (30–150 °C). The rapid heating rate of 5 °C min^−1^ makes it that the absorbed water in the coconut shell is difficult to be thoroughly released before 100 °C in time. A cliff-like mass decrease of 75.82% is ascribed to the elimination of pyrolysis products via the disintegration of bio-mass molecules at the green middle-temperature range of 150–600 °C. Further carbonization causes the TG curve to steadily diminish and reach a plateau at the last bule high-temperature area between 600 and 900 °C, leaving 15.75% of the original mass. Figure 1b shows the XRD patterns and illustrates the phase formation of all samples. The absence of prominent potassium compound signals suggests that the carbon samples were successfully rinsed with diluted HCl and deionized water to bring them to neutrality. At around 23° and 43°, there are two large diffraction peaks that are attributed to the (002) and (100) planes of amorphous carbon. The near graphitization degree is certified by the lack of substantial difference between the diffraction peaks of CSAC and CSC. Four Gaussian–Lorentzian peaks are fitted in Raman spectra (Figure 1c) to better compare the graphitization degree: I band observed at 1220 cm^−1^ is ascribed to the impurities near carbon atoms, D band around 1355 cm^−1^ is attributed to the breathing mode of *sp*^2^-hybridezed structure units which is active in the presence of defects, Dʹ band positioned around 1490 cm^−1^ is caused by the defects from stacked graphene layers and G band, located at 1590 cm^−1^, is connected to the vibration of graphitic *sp*^2^-type carbon [32,33]. A higher degree of graphitization caused by KOH activation is indicated by a lower *I_D_*/*I_G_* value (1.50) obtained from CSAC, as assessed by comparing the peak area ratio of D band and G band (*I_D_*/*I_G_*). In addition, the CSC and CSAC both have the obvious 2D bands around 2700 cm^−1^, indicating the existence of layer graphene. The amorphous-like structure of the activated carbon derived from coconut shell is shown by the results of the XRD pattern and Raman spectra discussed above.

**Figure 2:**
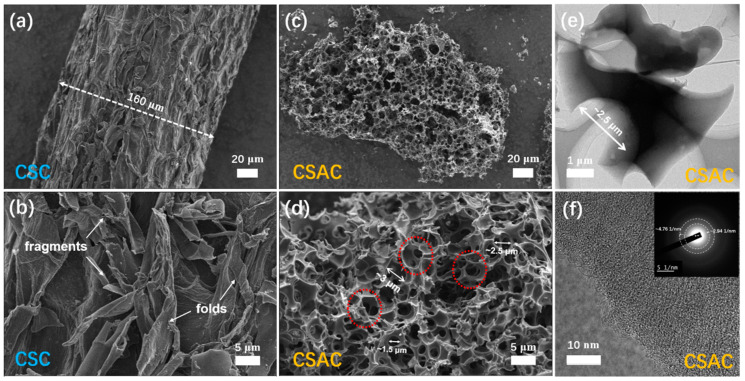
SEM images of (**a**,**b**) CSC and (**c**,**d**) CSAC, (**e**,**f**) TEM images of CSAC at different magnifications and diffraction fringes in the selected area electron diffraction.

The SEM and TEM images in Figure 2 show the micro-morphologies and general pore structure of CSC and CSAC. As depicted in Figure 2a, CSC has a surface covered in rough fragments or folds, and its coarse fibers have a diameter of 160 μm (Figure 2b). The optical microscope was also used to inspect the coconut shell to illustrate how fibrous it was prior to a high-temperature treatment (Figure S1). Figure 2c for the CSAC sample shows a honeycomb-like feature with a clear porosity structure following a one-step KOH activation at 800 °C for 2 h. In Figure 2d, the linked pores that the CSAC possesses (depicted by red dotted circles) with an average diameter of ~2.5 μm are visible when closer inspection is performed. The distinctive porosity architecture and the size of diameter were further evidenced by the TEM image (Figure 2e). The high-resolution TEM image (Figure 2f) also showed the presence of the micro- and mesopores as a disorder structure. There is no obvious lattice fringe in the magnified SAED (inset of Figure 2f), further substantiating the amorphous state of CSAC. In addition, the unclear concentric rings in the SAED pattern are estimated with the radius of ~4.76 and 2.94 1/nm, which are connected to the (100) and (002) planes, respectively. Herein, KOH activation proves effective in inducing a honeycomb-like morphology with interconnected macropores in coconut shell-derived carbon [23,34]. This network of interconnected macropores serves to facilitate electrolyte ions into the mesopores/micropores, thereby expediting their access to the carbon materials surfaces. This mechanism results in the highly efficient use of the substantial specific surface area, leading to an enhancement in the rate performance.

**Figure 3:**
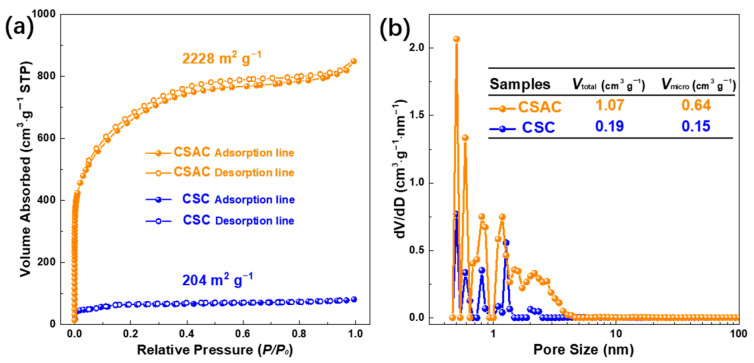
(**a**) N_2_ physisorption isotherms and (**b**) pore size distributions of CSC and CSAC.

The N_2_ physisorption isotherms of CSC and CSAC are presented in Figure 3a. Initially, it appears that both CSC and CSAC possess copious micropores leveraging the steep absorption of nitrogen under the relative pressure of 0.05. Additionally, the middle relative pressure region of the CSAC isotherm exhibits greater adsorption and desorption platforms, indicating that KOH activation increases the CSAC sample’s specific surface area to 2228 m^2^ g^−1^. According to IUPAC classification, the adsorption–desorption isotherm of CSC appears to be type-I curve, whereas the CSAC isotherm exhibits type-IV features with a typical H2 hysteresis loop in the relative pressure range of 0.4–1.0. In CSAC, such a hysteresis loop is assigned to a mesopore feature, which is further verified by the pore size distribution. Following KOH activation, there is a noticeable increase in pore volume within the range of 0.4 to 5 nm, as seen in Figure 3b. Delving into further detail, the values tabulated in Table 1 indicate an increase in total pore volumes and micropore volumes from 0.19 and 0.15 cm^3^ g^−1^ for CSC to 1.07 and 0.64 cm^3^ g^−1^ for CSAC. This enhancement can be primarily ascribed to the activation reaction of KOH with coconut shell at elevated temperatures. This process not only generates a greater number of micropores but also fosters the evolution of these micropores into mesopores. Consequently, such micro-, meso- and macropores characterized by N_2_ physisorption and SEM together formed the honeycomb-like hierarchical porous architecture, which facilitates swift ion transportation from the electrolyte to the adsorption site on the surface of internal micropores.

**Figure 4:**
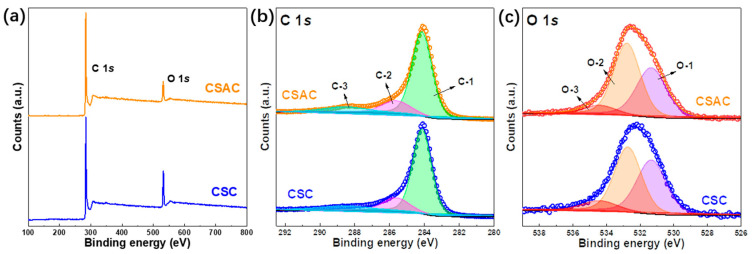
(**a**) XPS survey spectra of CSC and CSAC. High-resolution spectra of (**b**) C 1*s* and (**c**) O 1*s*.

The wide-scan XPS survey spectra, as depicted in Figure 4a, confirm the main composition of the C (284.5 eV) and O (531.6 eV) species in CSC and CSAC samples, which is attributed to the predominance of cellulose and lignin in the coconut shell. As evident in Table 1, the surface status was noticeably affected by the KOH activation. As a result of this KOH activation at a high temperature, there is a substantial increase in the C content, from 81.02 at.% for CSC to 88.08 at.% for CSAC. The high-resolution spectra are further presented in Figure 4b for C 1*s* and Figure 4c for O 1*s*. High-resolution C 1*s* spectra exhibit three resolved peaks located at 284.4, 285.7 and 288.5 eV, correspondingly associated with the C−C/C=C (C-1), C−O (C-2) and O−C=O (C-3) groups [35]. In terms of the deconvoluted O 1*s* region, three fitted peaks are distinguished at 531.4 eV, 532.6 eV and 533.6 eV, which are credited to quinone oxygen (O-1), phenol groups (O-2) and carboxyl groups (O-3) [36]. Even though the oxygen content is not as abundant as in CSC, CSAC exhibits more exposed oxygen sites on its surface due to an ample specific surface area (2228 m^2^ g^−1^). Such oxygen groups on the surface effectively hasten the electrochemical storage activities by enabling electron transfer and providing additional pseudocapacitance.

**Figure 5:**
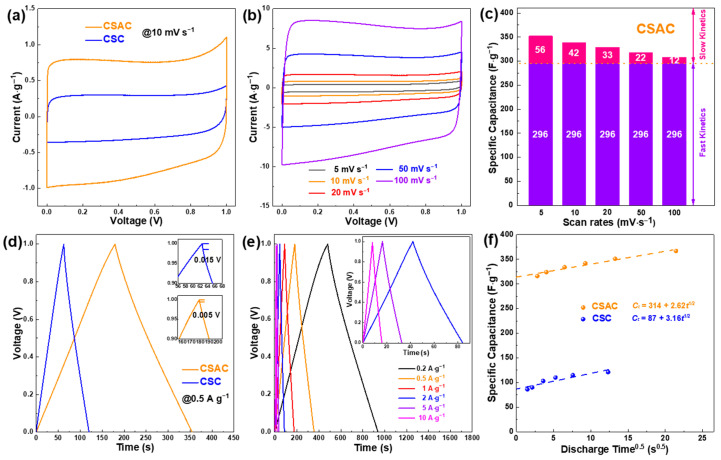
Electrochemistry characterizations of CSC and CSAC electrodes tested in the 6 M KOH symmetric supercapacitor: (**a**) CV curves at 10 mV s^−1^, (**b**) CV curves of CSAC at various scan rates, (**c**) histogram of the decoupling capacitance contributions of CSAC at various scan rates, (**d**) GCD curves at 0.5 A g^−1^, (**e**) GCD curves of CSAC at various current densities, (**f**) specific capacitance vs. square root of discharge time.

The electrochemical performances of pyrolyzed products are initially appraised in the symmetric supercapacitor in 6 M KOH electrolyte at 0–1.0 V. CV profiles (Figure 5a) and at 10 mV s^−1^ display a quasi-rectangular shape within both the CSC and CSAC electrodes, implying ideal capacitive performance. In addition, CSAC displays a more extensive CV profile area than CSC, indicating a higher specific capacitance achieved. Figure 5b exhibits that the CV profiles of the CSAC electrode nearly maintain the rectangular-like shapes from 5 to 100 mV s^−1^, illustrating excellent reproducible capacitive behavior. To delve further into the charge storage kinetics, CV curves of CSC and CSAC were explored at various scan rates, as in the following Equation (1):

(1) $$i=k_{1}v+k_{2}v^{1/2}$$

where *k*_1_*v* refers to the current density related to a fast kinetic response, which are primarily surface-dominated, *k*_2_*v*^1/2^ equals the current density resulting from slow kinetic processes, which is mainly associated with the diffusion of ions. As plotted in Figure 5c and Figure S2, the devices of CSC and CSAC exhibit a fast kinetic capacitance of 90 and 296 F g^−1^, respectively. The GCD curves of CSC- and CSAC-based devices, performed at 0.5 A g^−1^ (Figure 5d), exhibit a triangle-like distribution with a small Ohmic drop, implying optimal electrical double-layer capacitive characteristics. Furthermore, the GCD curves of CSAC-based devices are distinguished by isosceles triangles and good linearity at current densities from 0.2 to 10 A g^−1^ (Figure 5e), which align well with CV results. The specific capacitance of a CSAC electrode, derived from discharge curves, reveals an exceptionally high value of 367 F g^−1^ at 0.2 A g^−1^ and retains 316 F g^−1^ at 10 A g^−1^. The electrochemical properties of both CSC and CSAC electrodes, derived from CV and GCD curves under different scan rates/current densities, are tabulated in Table S1. As depicted in Figure 5f, the electrical double-layer capacitance (*C_E_*) calculated from the intercept with a vertical coordinate gives 314 F g^−1^ for CSAC and 87 F g^−1^ for CSC, respectively. A detailed comparison of *C_t_* (the total electrochemical capacitance), *C_E_* and *C_p_* (pseudocapacitance) are tabulated in Table 1.

**Figure 6:**
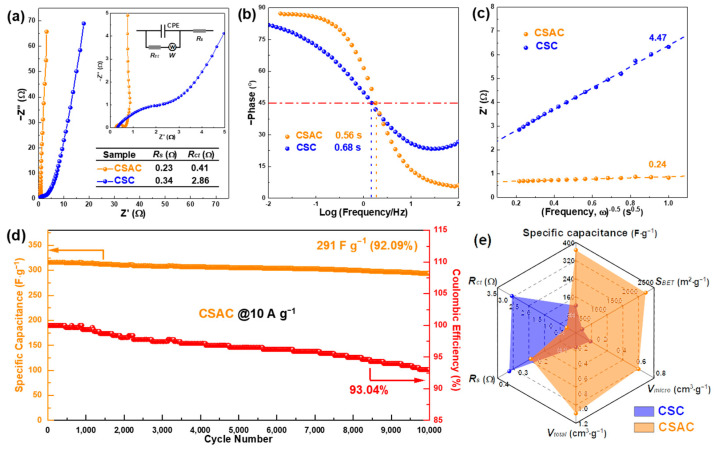
Electrochemical properties of CSC and CSAC electrodes: (**a**) Nyquist plots with frequency range of 10^5^-0.01 Hz, (**b**) Bode plots, (**c**) Randles plots, (**d**) cycling stability of CSAC at 10 A g^−1^ and (**e**) the comparison Radar chart of six parameters.

The Nyquist diagrams in Figure 6a illustrate that the CSAC-based device achieves a much lesser equivalent series resistance (*R_s_*) of 0.23 Ω as well as charge transfer resistance (*R_ct_*) of 0.41 Ω than the CSC-based device. According to the related Bode plots (Figure 6b) and Randles plots (Figure 6c), the relaxation time constant *τ* value and diffusive resistance σ value of a CSAC-based device is 0.59 s and 0.24 Ω s^−0.5^, respectively, indicating a quick frequency response and swift access of ions to the internal interaction site. The long-term cycle life of a CSAC-based device, shown in Figure 6d, reveals excellent electrochemical stability. The capacitance has decayed from 316 F g^−1^ to 291 F g^−1^ (retention of 92.09% with coulombic efficiency of 93.04%) at the high current density of 10 A g^−1^ after 10,000 cycles, highlighting superior reversibility. Figure 6e presents the Radar chart containing six parameters (specific capacitance, *S_BET_*, *V_total_*, *V*_micro_, *R_s_* and *R_ct_*) for a clear comparison of the CSC and CSAC samples. Table S2 tabulates the electrochemical properties of CSAC and other biomass-derived carbons-based symmetric supercapacitors. Reflecting upon all these results, the outstanding performance of CSAC can be mainly ascribed to one-step KOH activation, which not only generates the honeycomb-like morphology with a hierarchical porous structure, enhancing the exposure degree of a large surface area with more O active sites, but it also induces a high graphitization with low disordering, which in turn improves the charge carrier transport and conductivity.

**Figure 7:**
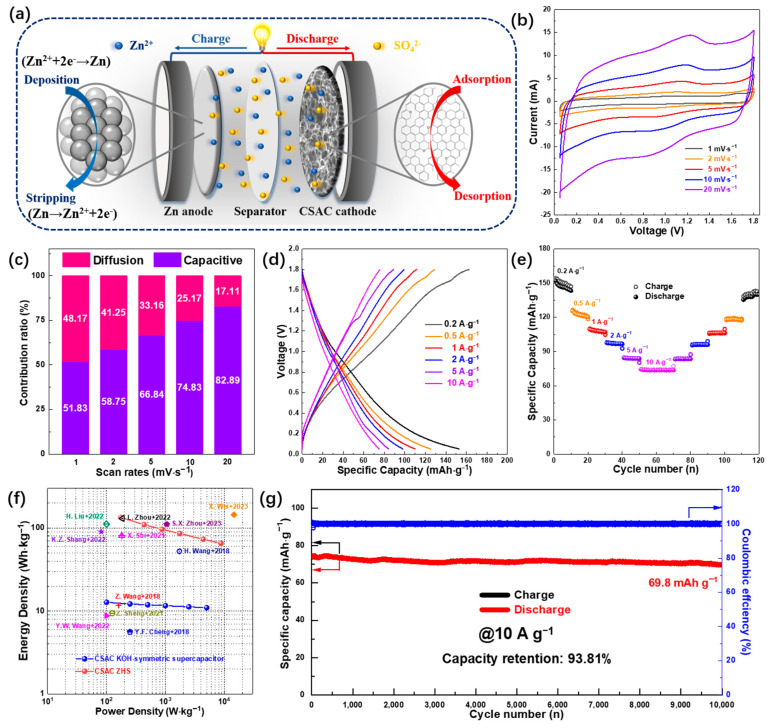
(**a**) Configuration and working mechanism of ZHS. Electrochemical performance of ZHS based on CSAC cathode, (**b**) CV curves at various scan rates, (**c**) the capacitive and diffusion contribution ratios to the total capacity at various scan rates, (**d**) galvanostatic charge–discharge profiles at various current densities, (**e**) rate performance, (**f**) Ragone plots [14,37,38,39,45,46,47,48,49,50,51] and (**g**) cycling performance at 10 A g^−1^.

The as-prepared CSAC exhibits a hierarchical porous structure, large specific surface area and high electrical conductivity, which all foster greater acceptability and a more concise transfer pathway for electrolyte ions and electrons. Encouraged by the above remarkable characteristics and the small hydrated radii of SO_4_^2−^ (3.79 Å), an aqueous ZHS device (Zn//ZnSO_4_(*aq.*)//CSAC) is further assembled to probe into its practical application. Figure 7a depicts the working principle of a ZHS device. Benefitting from the integration of a battery-type anode and supercapacitor-type cathode, the energy storage in the ZHSs is mainly through the reversible Zn^2+^ deposition/stripping onto the Zn anode as well as through the anion adsorption/desorption on the surface of the CSAC cathode. Therefore, all electrochemical results reported in Figure 7b–g confirm that a ZHS device may attain the noted energy and power densities. As depicted in Figure 7b, the CV curves of the ZHS device show no peaks in oxygen and hydrogen generation at various scan rates, indicating that the ZHSs can operate well from 0.05 to 1.8 V. Furthermore, these undesirable rectangular profiles illustrate a varied electrochemical performance of the CSAC cathode and Zn anode in a ZHS device. Simultaneously, the preceding Equation (1) was used to quantify the different process-controlled contributions of the ZHSs, where *k_1_v* refers to capacitive contribution and *k*_2_*v*^1/2^ denotes the diffusion contribution. As illustrated in Figure 7c and Figure S3, the capacitive-driven process provides about 52.83% of the total storage capacity at the scan rate of 1 mV s^−1^. As the scan rate increased to 20 mV s^−1^, there was a gradual uptick in the capacitive contribution ratio to 82.89%, while the diffusion contribution ratio correspondingly declined to 17.11%. This change validates the capacitive-dominant nature from the CSAC cathode and expedited electrochemical kinetics at a high scan rate. GCD curves were applied to determine the specific capacity at varying current densities (Figure 7d). Based on the calculation, the discharge specific capacities of the ZHSs, when employing the CSAC cathode, reach 153, 125, 110, 98, 84 and 75 mAh g^−1^ at current densities of 0.2, 0.5, 1, 2, 5 and 10 A g^−1^. This performance led to a capacity retention of 49%. Encouragingly, as depicted in Figure 7e, the capacity in terms of rate performance reverted to 140 mAh g^−1^ after 120 cycles at a reset current of 0.2 A g^−1^, indicating an excellent reversibility of the ZHSs. Additionally, near-linearity without a notable potential plateaus feature in GCD curves suggests both electrochemical double-layer capacitive and pseudocapacitive mechanisms in a hybrid device. Figure 7f displays the Ragone plots for both symmetric and hybrid supercapacitors. The KOH-loaded symmetric device of CSAC exhibits an energy density of 12.75 Wh kg^−1^ at a power density of 100 W kg^−1^ [37,38,39]. Inspired by the outstanding capacity and wide potential window, a greater energy density of 134.9 Wh kg^−1^ was achieved by the ZHSs at a power density of 175 W kg^−1^. Even more specifically, CSAC-based ZHSs still retained an energy density of 62.6 Wh kg^−1^ at an ultrahigh power density of 8750 W kg^−1^. As presented in Figure 7g, a high specific capacity of 70 mAh g^−1^ was obtained at 10 A g^−1^ after 10,000 cycles, coupled with 93.81% capacity retention and high coulombic efficiency. Nyquist plots of CSAC-based ZHSs depicted in Figure S4 exhibit relatively low *R_s_* (2.23 Ω) and *R_ct_* (10.22 Ω) values. The exceptional electrochemical performance of the CSAC cathode-based ZHS device, in comparison to various ZHS devices as reported in Table 2, can be primarily attributed to the unique characteristics of a CSAC [40,41,42,43,44,45,46,47,48,49,50,51]. This includes its honeycomb-like morphology, hierarchical porous structure, large specific surface area, suitable O content and high conductivity, which collectively enhance ion diffusion and electron transport.

## 3. Materials and Methods

### 3.1. Preparation of Coconut Shell-Derived Activated Carbon

**Figure 8:**
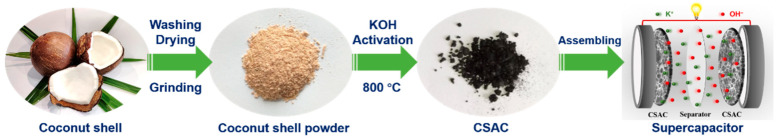
Schematic diagram for preparing CSAC and its application in supercapacitors.

Coconut shells were sourced from a local fruit market near Jiujiang University. Initially, these shells were chopped into small fragments and thoroughly cleaned to remove the residual dust. The fragments were then oven-dried at 60 °C for 48 h, after which they were ground into powder. This obtained coconut shell powder was mixed with KOH in a weight ratio of 1:1, and then activated at 800 °C for 2 h under a nitrogen atmosphere, maintaining a heating rate of 2 °C min^−1^. The resulting mixture was washed with dilute HCl and deionized water until it reached a pH of 7, and then oven-dried overnight at 60 °C to yield the coconut shell-derived activated carbon (denoted as CSAC). For comparison, the coconut shell-derived carbon (abbreviated as CSC) was also prepared without adding KOH, following the same procedure. Figure 8 provides a schematic diagram depicting the CSAC preparation process and its subsequent application in supercapacitors.

### 3.2. Characterization

An STA800 thermogravimetric analyzer (PerkinElmer, Waltham, MA, USA) was employed to perform the thermogravimetric analysis of coconut shell from room temperature to 900 °C under nitrogen with a heating rate of 5 °C min^−1^. The compositional phases and crystallographic structure of the carbon samples were examined by the XRD patterns generated by a Bruker Focus D8 Advance diffractometer (Karlsruhe, Germany) with Cu Kα radiation (λ = 1.54 Å). Raman spectra were procured with a Renishaw Inviaspectrometer (London, British), using an excitation wavelength of 532 nm, an excitation power of 1.5 mW and an acquisition time of 55 s. The surface structures and morphology characterizations of carbon samples were visualized by an S-4800 field-emission scanning electron microscope (SEM, Tokyo, Japan) and JEOL JEM-2100 transmission electron microscope (TEM, Tokyo, Japan), while the coconut shell was observed with an SZ810 optical microscope (OPTEC, Chongqing, China). The samples were degassed at 200 °C for 2 h, and then tested on a Micromeritics ASAP 2460 analyzer (Norcross, GA, USA) at −196 °C to obtain the nitrogen physisorption isotherms. The specific surface areas were evaluated from the Brunauer–Emmett–Teller (BET) method, and the pore size distributions were calculated based on the nonlocal density functional theory (DFT) model. The surface chemical compositions were detected by an AXIS Ultra DLD X-ray photoelectron spectrometer (XPS, Shimadzu, Kyoto, Japan) equipped with Al Kα radiation. All samples charging were calibrated using the C 1s peak (284.4 eV) as an internal standard.

### 3.3. Electrochemical Measurements

All electrochemical performances of carbon samples were evaluated using CR2032 coin-type cells. For symmetric supercapacitors, a homogeneous slurry was created by combining the as-prepared carbon, acetylene black and polytetrafluoroethylene (PTFE) in ethanol and N-methyl-2-pyrrolidinone (NMP) solution at a ratio of 8:1:1. The working electrode was then created by pressing the dry slurry under 15 MPa on nickel foam. A single electrode had approximately 2 mg cm^−2^ active material. Two identical working electrodes were assembled in the 6 M KOH-loaded symmetric supercapacitor, where a piece of filter paper was applied as a separator. The electrochemical measurements, comprising cyclic voltammetry (CV), galvanostatic charge–discharge (GCD) and electrochemical impedance spectroscopies (EIS), were conducted on a coin-type device using a Chenhua electrochemical workstation (CHI660D, Shanghai, China). The specific capacitance (*C*, F g^−1^) of the individual electrode in the symmetric supercapacitor was determined based on the CV and GCD curves, according to the following Equations (2) and (3):

(2) $$C_{CV}=4\times \frac{\int^{\;}idV}{2\times \Delta V\times m\times r}$$

(3) $$C_{GCD}=4\times \frac{I\times \Delta t}{\Delta V\times m}$$

where Δ*V* (V) is the operation potential window, *r* (V s^−1^) is the scan rate, *m* (g) is the total mass of both electrodes and Δ*t* (s) is the discharging time. *i* (A) in Equation (2) and *I* (A) in Equation (3) is the response current and the discharging current, respectively.

The ZHSs were also assembled in a CR2032 coin cell, employing zinc foil (12 mm in diameter), 1 M ZnSO_4_ solution and a glass fiber membrane as the anode, the electrolyte and the separator, respectively. The CSAC cathode electrode included a CSAC powder (80 wt.%), conductive acetylene black (10 wt.%) and poly(vinylidene fluoride) (PVDF, 10 wt.%). N-methyl-2-pyrrolidinone (NMP) solution was added to this mixture to form a uniform slurry. This was then coated on stainless steel foil and subsequently dried in a vacuum oven at 60 °C for 24 h. The CV and EIS measurements of ZHS were conducted on a Chenhua electrochemical workstation, while the GCD and long circles tests were evaluated by a CT2001 battery testing system (LAND, Wuhan, China).

## 4. Conclusions

In summary, we have successfully synthesized a hierarchical porous activated carbon from coconut shells via one-step KOH activation. The resultant CSAC materials feature exceptional properties including a honeycomb-like morphology, hierarchical porous structure, large specific surface area and commendable electrical conductivity. Thanks to these distinct advantages, a CSAC electrode manifests superior electrochemical performances in symmetric and hybrid supercapacitors. An ultrahigh specific capacitance of 367 F g^−1^ was achieved by a CSAC electrode in a 6 M KOH loaded-symmetric supercapacitor, maintaining 92.09% capacity retention even after 10,000 cycles at 10 A g^−1^. More notably, the ZHSs using CSAC as a cathode achieve a high-rate capability (153 mAh g^−1^ at 0.2 A g^−1^ and 75 mAh g^−1^ at 10 A g^−1^), high energy density (134.9 Wh kg^−1^ at 175 W kg^−1^), as well as outstanding cycling stability (93.81% capacity retention after 10,000 cycles at 10 A g^−1^). Possessing excellent energy storage performance affirms that coconut shell-derived HPAC is a prospective candidate in ultrahigh-performance supercapacitors for industrial large-scale applications.

## Figures and Tables

**Table 1:** Pore characteristics, surface elemental compositions and capacitances of CSC and CSAC ^a^.

Samples	*S*_BET_ (m^2^ g^−1^)	*V*_total_ (cm^3^ g^−1^)	*V*_micro_ (cm^3^ g^−1^)	C (at.%)	O (at.%)	*C*_t_ (F g^−1^)	*C*_E_ (F g^−1^)	*C*_P_ (F g^−1^)
CSC	204	0.19	0.15	81.82	18.18	121	87	34
CSAC	2228	1.07	0.64	88.08	11.92	367	314	53

^a^ *S*_BET_, specific surface area; *V*_total_, the total pore volume calculated from the Density Functional Theory (DFT) method; *V*_micro_, the pore volume of the micropores; *C*_t_, the total electrochemical capacitance at 0.2 A g^−1^ tested in two-electrode system using KOH electrolyte; *C*_E_, electric double-layer capacitance; *C*_P_, pseudocapacitance.

**Table 2:** Electrochemical performance comparison of aqueous ZHSs based on different cathodes ^b^.

Cathode Materials	Electrolyte	*V* (V)	*C_lc_* (mAh g^−1^)	*C_hc_* (mAh g^−1^)	*E* (Wh kg^−1^)	Ref.
CSAC	1 M ZnSO_4_	0.05–1.8	152 (0.2 A g^−1^)	75 (10 A g^−1^)	134.9	This work
BN-LDC	1 M ZnSO_4_	0.2–1.8	127.7 (0.5 A g^−1^)	42.8 (10 A g^−1^)	97.6	[40]
PANI	2 M ZnCl_2_	0.7–1.7	142.3 (0.2 A g^−1^)	81.1 (4 A g^−1^)	117.5	[41]
AC	2 M ZnSO_4_	0.2–1.8	121.0 (0.1 A g^−1^)	41.0 (1 A g^−1^)	84	[42]
OPCNF-20	1 M ZnSO_4_	0.2–1.8	136.4 (0.1 A g^−1^)	38.7 (20 A g^−1^)	97.7	[43]
HNPC	1 M ZnSO_4_	0–1.8	177.8 (4.2 A g^−1^)	108.2 (33.3 A g^−1^)	107.3	[44]
TFMA	2 M ZnSO_4_	0.1–1.8	107.0 (1 A g^−1^)	53 (10 A g^−1^)	110.8	[45]
C-0.6	2 M ZnSO_4_	0.2–1.8	181.7 (0.05 A g^−1^)	66.7 (20 A g^−1^)	145.2	[46]
HPCS-900	2 M ZnSO_4_	0.1–1.7	104.7 (0.1 A g^−1^)	40.2 (20 A g^−1^)	90.2	[47]
CSGC	2 M ZnSO_4_	0.2–1.8	138.8 (0.1 A g^−1^)	85.6 (20 A g^−1^)	111.1	[48]
N-HHPC	2 M ZnSO_4_	0.1–1.8	140.7 (0.2 A g^−1^)	101.3 (100 A g^−1^)	130.2	[49]
AC-CS	1 M Zn(CF_3_SO_3_)	0–1.8	85.7 (0.1 A g^−1^)	38.1 (2 A g^−1^)	52.7	[50]
NPC	1 M ZnSO_4_	0–1.8	136.2 (0.3 A g^−1^)	69.2 (15 A g^−1^)	81.1	[51]

^b^ *V*, the practical working voltage; *C_lc_*, the specific capacity at low specific current; *C_hc_*, the specific capacity at high specific current; *E*, the energy density.

## Data Availability

Not applicable.

## Supplementary Materials

The following supporting information can be downloaded at: https://www.mdpi.com/article/10.3390/molecules28207187/s1. Refs. [52,53,54,55,56,57,58,59,60,61,62,63,64,65,66] are cite in the Supplementary Materials.
