# Supplementary material for: Hierarchical Porous Activated Carbon Derived from Coconut Shell for Ultrahigh-Performance Supercapacitors
Source: Molecules. 2023 Oct 20;28(20):7187. doi: 10.3390/molecules28207187 (PMC10609479; doi:10.3390/molecules28207187)
Supplement: Supplementary file 1 [file molecules-28-07187-s001.zip › molecules-2630001-supplementary.pdf]

Supporting Information

Hierarchical Porous Activated Carbon Derived from Coconut Shell for Ultrahigh-Performance Supercapacitors

Yawei Wang [1,*], Yuhui Duan [2], Xia Liang [1], Liang Tang [1], Lei Sun [1], Ruirui Wang [1], Shunhang Wei [3], Huanan Huang [1], Pinghua Yang [1] and Huanan Hu [1,*]

1 School of Chemistry and Chemical Engineering, Jiangxi Province Engineering Research Center of Ecological Chemical Industry, Jiujiang University, Jiujiang 332005, China; 20200205648@jju.edu.cn (X.L.); 20200200942@jju.edu.cn (L.T.); 20200205239@jju.edu.cn (L.S.); 6080129@jju.edu.cn (R.W.); huanghn@jju.edu.cn (H.H.); 6080021@jju.edu.cn (P.Y.)

2 Institute for Advanced Interdisciplinary Research (iAIR), University of Jinan, Jinan 250022, China; 202130222057@stu.ujn.edu.cn

3 School of Mathematical Information, Shaoxing University, Shaoxing 312000, China; wsh@usx.edu.cn

* Correspondence: wangyw@jju.edu.cn (Y.W.); 6080075@jju.edu.cn (H.H.)

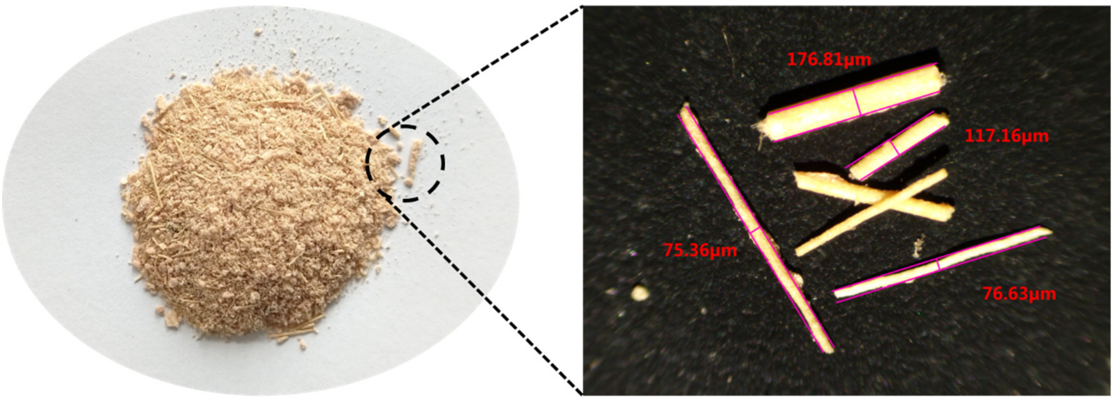

Figure S1. The optical micrograph of coconut shell powder.

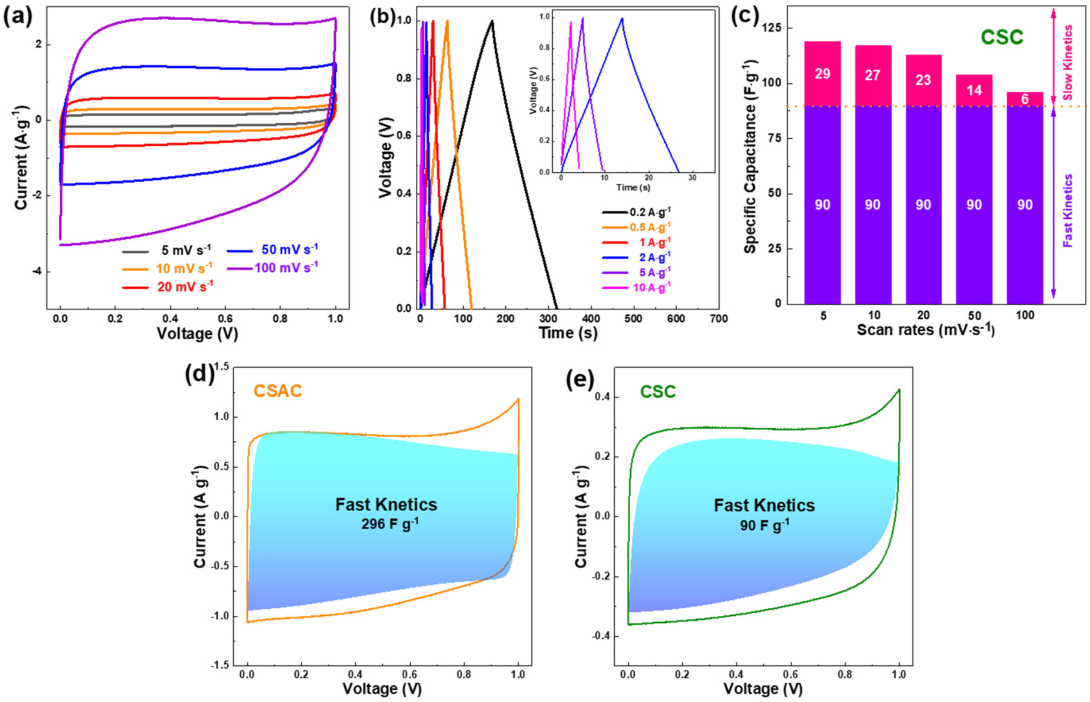

Figure S2. (a) CV and (b) GCD curves of CSC electrode at various scan rates or current densities. (c) Histogram of the decoupling capacitance contributions of CSC at various scan rates. Decoupling capacitance of (c) CSAC and (d) CSC electrode contributed by the fast-kinetic process (blue area) and the slow-dynamic processes (blank) at 10 mV s^{-1}.

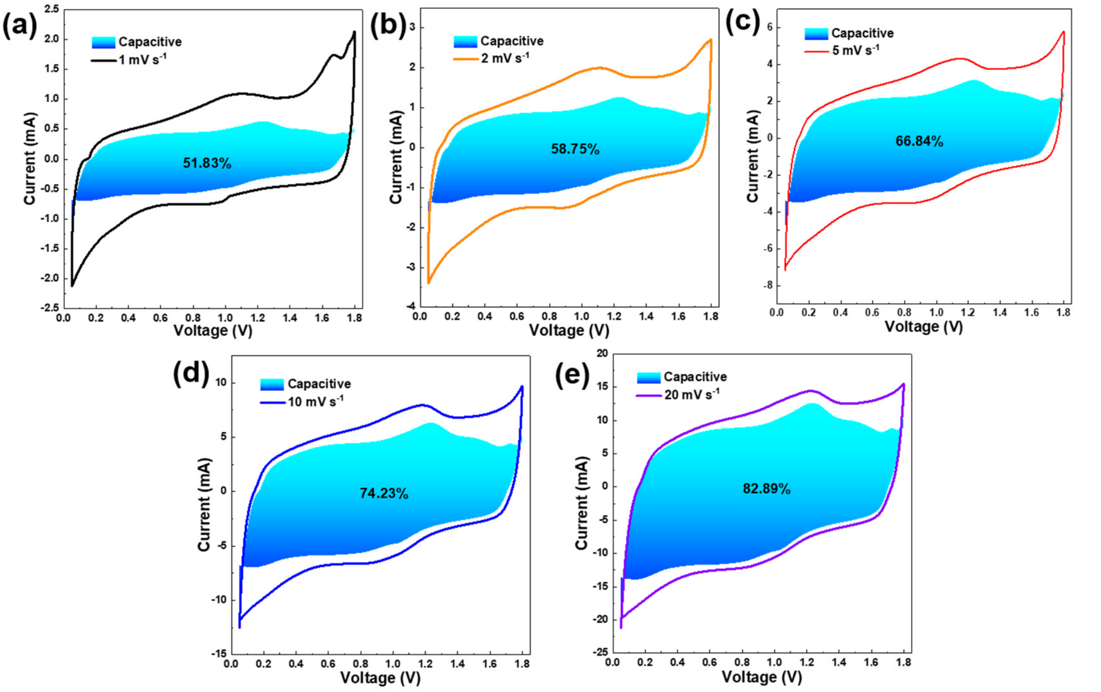

Figure S3. Capacitive contributions of CSAC cathode in ZHS at various scan rates: (a) 1 mV s^{-1}, (b) 2 mV s^{-1}, (c) 5 mV s^{-1}, (d) 10 mV s^{-1} and (e) 20 mV s^{-1}.

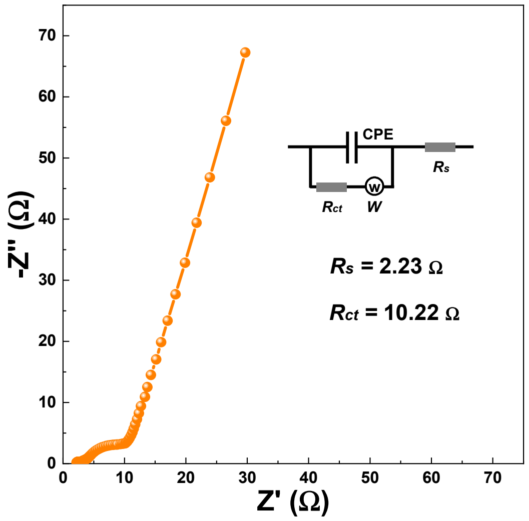

Figure S4. Nyquist plots of CSAC-based ZHS with frequency range of 10^5-0.01 Hz.

Table S1. Electrochemical capacitances of CSC and CSAC electrodes calculated from CV and GCD curves of 6 M KOH-loaded symmetric device.

Sample	Capacitance calculated from CV curves at various scan rates				
	5 mV s^{-1}	10 mV s^{-1}	20 mV s^{-1}	50 mV s^{-1}	100 mV s^{-1}
CSC	119 F g^{-1}	117 F g^{-1}	113 F g^{-1}	104 F g^{-1}	96 F g^{-1}
CSAC	354 F g^{-1}	340 F g^{-1}	331 F g^{-1}	320 F g^{-1}	311 F g^{-1}

Sample	Capacitance calculated from GCD curves at various current densities					
	0.2 A g^{-1}	0.5 A g^{-1}	1 A g^{-1}	2 A g^{-1}	5 A g^{-1}	10 A g^{-1}
CSC	121 F g^{-1}	115 F g^{-1}	110 F g^{-1}	103 F g^{-1}	90 F g^{-1}	86 F g^{-1}
CSAC	367 F g^{-1}	351 F g^{-1}	343 F g^{-1}	334 F g^{-1}	324 F g^{-1}	316 F g^{-1}

Table S2. Electrochemical performance comparison of CSAC- and other biomass-derived carbons-based symmetric supercapacitors.

Precursor	Sample	Electrolyte	Capacitance (F g^{-1})	Capacitance retention	Cycling stability	Ref.
Coconut shell	CSAC	6 M KOH	367 (0.2 A g^{-1})	86.10% (0.2-10 A g^{-1})	92.09% (10 A g^{-1}, 10000 cycles)	This work
Coconut shell	3DPGLS	1 M TEMABF4/PC	91.15 (0.2 A g^{-1})	89.2% (0.2-2 A g^{-1})	85.1% (0.1 A g^{-1}, 5000 cycles)	[1]
Coconut shell	CSCK-800-2	6 M KOH	317 (0.5 A g^{-1})	68% (0.5-20 A g^{-1})	99.7% (5 A g^{-1}, 10000 cycles)	[2]
Coconut shell and coal	CJA15	6 M KOH	251.67 (0.5 A g^{-1})	77.9% (0.5-10 A g^{-1})	-	[3]
Durian kernel	DKAC-700	6 M KOH	330 (0.2 A g^{-1})	75.15% (0.2-20 A g^{-1})	97.9% (2 A g^{-1}, 10000 cycles)	[4]
Pistachio shell	NCNaK-1	1 M KOH	88 (0.5 A g^{-1})	77.27% (0.5-5 A g^{-1})	95.8% (10 A g^{-1}, 5000 cycles)	[5]
Corn husk	ASCH-1:1	6 M KOH	127 (1 A g^{-1})	69.0% (1-20 A g^{-1})	-	[6]
Plastic bags	PE-HPC-900-NH_3	6 M KOH	110 (0.05 A g^{-1})	90.9% (0.05-0.5 A g^{-1})	97.1% (2 A g^{-1}, 10000 cycles)	[7]
Feather finger grass flower	HT-PC	6 M KOH	120 (1 A g^{-1})	70.6% (1-30 A g^{-1})	70% (10 A g^{-1}, 10000 cycles)	[8]
Bamboo	ABC-900	3 M KOH	79 (0.5 A g^{-1})	81.9% (0.5-20 A g^{-1})	91.8% (3 A g^{-1}, 10000 cycles)	[9]
Cornstalk	HPCS-3	1 M H_2SO_4	413 (0.5 A g^{-1})	65.9% (0.5-10 A g^{-1})	92.6% (5 A g^{-1}, 20000 cycles)	[10]
Cotton stalk	FTMAC-4	1 M H_2SO_4	254 (0.2 A g^{-1})	83.1% (0.2-10 A g^{-1})	96% (1 A g^{-1}, 10000 cycles)	[11]
Rice straw	NMCSs@RSPC-1	6 M KOH	268 (1 A g^{-1})	80.2% (1-10 A g^{-1})	90% (5 A g^{-1}, 4000 cycles)	[12]
Lotus leaf	LLPC-800-1:3	6 M KOH	274 (1 A g^{-1})	88% (1-10 A g^{-1})	90% (5 A g^{-1}, 5000 cycles)	[13]
Rose	RAC-800-2	1 M Na_2SO_4	128.8 (0.5 A g^{-1})	72.3% (1-4 A g^{-1})	96% (5 A g^{-1}, 10000 cycles)	[14]
Rice husk	PCNS/RHC_8	6 M KOH	315 (0.1 A g^{-1})	60% (0.1-50 A g^{-1})	95.8% (5 A g^{-1}, 10000 cycles)	[15]
Tremella	THPC 1-24	6 M KOH	299.3 (0.5 A g^{-1})	83.6% (0.5-20 A g^{-1})	97% (10 A g^{-1}, 50000 cycles)	[16]

References

1. J.S. Xia, N. Zhang, S.K. Chong, D. Li, Y. Chen, C.H. Sun, Three-dimensional porous graphene-like sheets synthesized from biocarbon via low-temperature graphitization for a supercapacitor, Green Chem. 20 (2018) 694-700.
2. Y. Zhao, J. Mu, Y. Wang, Y. Liu, H. Wang, H. Song, Preparation of hierarchical porous carbon through one-step KOH activation of coconut shell biomass for high-performance supercapacitor, J. Mater. Sci-Mater. El. 34 (2023) 527.
3. M. Bora, D. Bhattacharjya, S. Hazarika, X. Fan, B.K. Saikia, Blending of activated low-grade coal powder with coconut shell waste for supercapacitor applications, Energ. Fuel. 36 (2022) 14476-14489.
4. Y. Wang, W. Lu, X. Xu, F. Pang, X. Feng, X. Zhang, Y. Zeng, Z. Yang, R. Wang, P. Yang, L. Chen, Ternary-doped hierarchical porous carbons derived from durian kernel as electrode materials for efficient energy storage devices, Diam. Relat. Mater. 130 (2022) 109451.
5. O.C. Altinci, M. Demir, Beyond conventional activating methods, a green approach for the synthesis of biocarbon and its supercapacitor electrode performance, Energ. Fuel. 34 (2020) 7658-7665.
6. M.U. Rani, K. Nanaji, T.N. Rao, A.S. Deshpande, Corn husk derived activated carbon with enhanced electrochemical performance for high-voltage supercapacitors, J. Power Sources 471 (2020) 228387.
7. Y.M. Lian, M. Ni, Z.H. Huang, R.J. Chen, L. Zhou, W. Utetiwabo, W. Yang, Polyethylene waste carbons with a mesoporous network towards highly efficient supercapacitors, Chem. Eng. J. 366 (2019) 313-320.
8. R.A. Senthil, V.K. Yang, J.Q. Pan, Y.Z. Sun, A green and economical approach to derive biomass porous carbon from freely available feather finger grass flower for advanced symmetric supercapacitors, J. Energy Storage 35 (2021) 102287.
9. G.X. Zhang, Y.M. Chen, Y.G. Chen, H.B. Guo, Activated biomass carbon made from bamboo as electrode material for supercapacitors, Mater. Res. Bull. 102 (2018) 391-398.
10. C.J. Wang, D.P. Wu, H.J. Wang, Z.Y. Gao, F. Xu, K. Jiang, A green and scalable route to yield porous carbon sheets from biomass for supercapacitors with high capacity, J. Mater. Chem. A 6 (2018) 1244-1254.
11. X. Tian, H.G. Ma, Z. Li, S.C. Yan, L. Ma, F. Yu, G. Wang, X.H. Guo, Y.Q. Ma, C.P. Wong, Flute type micropores activated carbon from cotton stalk for high performance supercapacitors, J. Power Sources 359 (2017) 88-96.
12. S.B. Liu, Y. Zhao, B.H. Zhang, H. Xia, J.F. Zhou, W.K. Xie, H.J. Li, Nano-micro carbon spheres anchored on porous carbon derived from dual-biomass as high rate performance supercapacitor electrodes, J. Power Sources 381 (2018) 116-126.
13. S.S. Qu, J.F. Wan, C.C. Dai, T.Y. Jin, F.W. Ma, Promising as high-performance supercapacitor electrode materials porous carbons derived from biological lotus leaf, J. Alloy. Compd. 751 (2018) 107-116.
14. C.J. Zhao, Y.X. Huang, C.H. Zhao, X.X. Shao, Z.Q. Zhu, Rose-derived 3D carbon nanosheets for high cyclability and extended voltage supercapacitors, Electrochim. Acta 291 (2018) 287-296.
15. S. Dong, X.J. He, H.F. Zhang, X.Y. Xie, M.X. Yu, C. Yu, N. Xiao, J.S. Qiu, Surface modification of biomass-derived hard carbon by grafting porous carbon nanosheets for high-performance supercapacitors, J. Mater. Chem. A 6 (2018) 15954-15960.
16. D. Chen, L. Li, Y.L. Xi, J.Z. Li, M.J. Lu, J.M. Cao, W. Han, Self-assembly of biomass microfibers into 3D layer-stacking hierarchical porous carbon for high performance supercapacitors, Electrochim. Acta 286 (2018) 264-270.
